# Tip‐Balloon Anchored Retroflex (T‐BAR) Method for Double Balloon Endoscopy‐Assisted ERCP in Roux‐en‐Y Anatomy

**DOI:** 10.1002/jhbp.70094

**Published:** 2026-02-23

**Authors:** Nobuhiko Fukuba, Erito Ando, Taisuke Ohmachi, Yasuhide Kodama, Masaki Onoe, Kousaku Kawashima, Norihisa Ishimura, Shunji Ishihara

**Affiliations:** ^1^ Department of Internal Medicine II Shimane University Faculty of Medicine Izumo Shimane Japan

## Abstract

With accompanying video, Fukuba and colleagues described the Tip‐Balloon Anchored Retroflex (T‐BAR) technique for double‐balloon enteroscopy‐assisted ERCP in Roux‐en‐Y anatomy with an intact papilla. By using the tip balloon as an anchor, the method stabilizes retroflex positioning, prevents scope slippage, improves biliary axis alignment, and facilitates selective biliary cannulation.

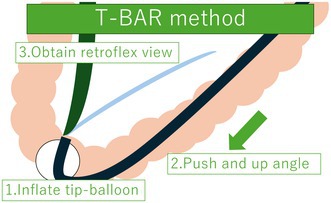

Double Balloon Endoscopy‐assisted ERCP (DBE‐ERCP) with a preserved papilla can fail when retroflex positioning is unstable and the catheter cannot be aligned coaxially with the bile duct [1, 2, 3, 4]. We describe the Tip‐Balloon Anchored Retroflex (T‐BAR) technique, a modified retroflex position technique, which uses the tip balloon of a double‐balloon endoscope as a fixation point (Figure 1).

**FIGURE 1:**
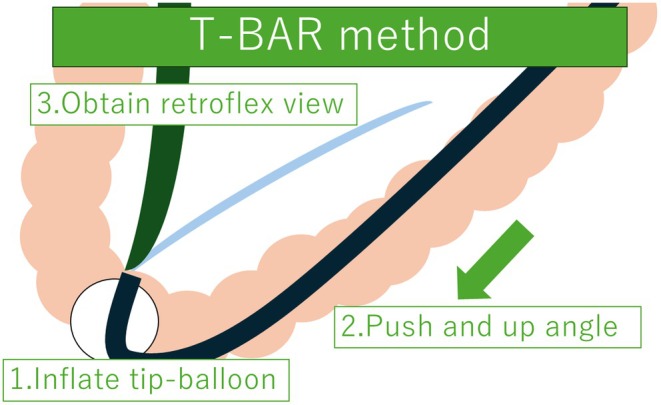
The concept of Tip‐Balloon Anchored Retroflex (T‐BAR) method.

## 1 Case (Video)

A man in his 70s with choledocholithiasis had undergone distal gastrectomy with Roux‐en‐Y reconstruction (Video S1). The papilla was visualized at the 6 o'clock position, but the bile duct axis was nearly perpendicular to the catheter axis and selective biliary cannulation failed. We attempted the retroflex position; however, the scope tip repeatedly slid toward the blind end, preventing stable positioning.

## 2 Technique (T‐BAR)

**FIGURE 2:**
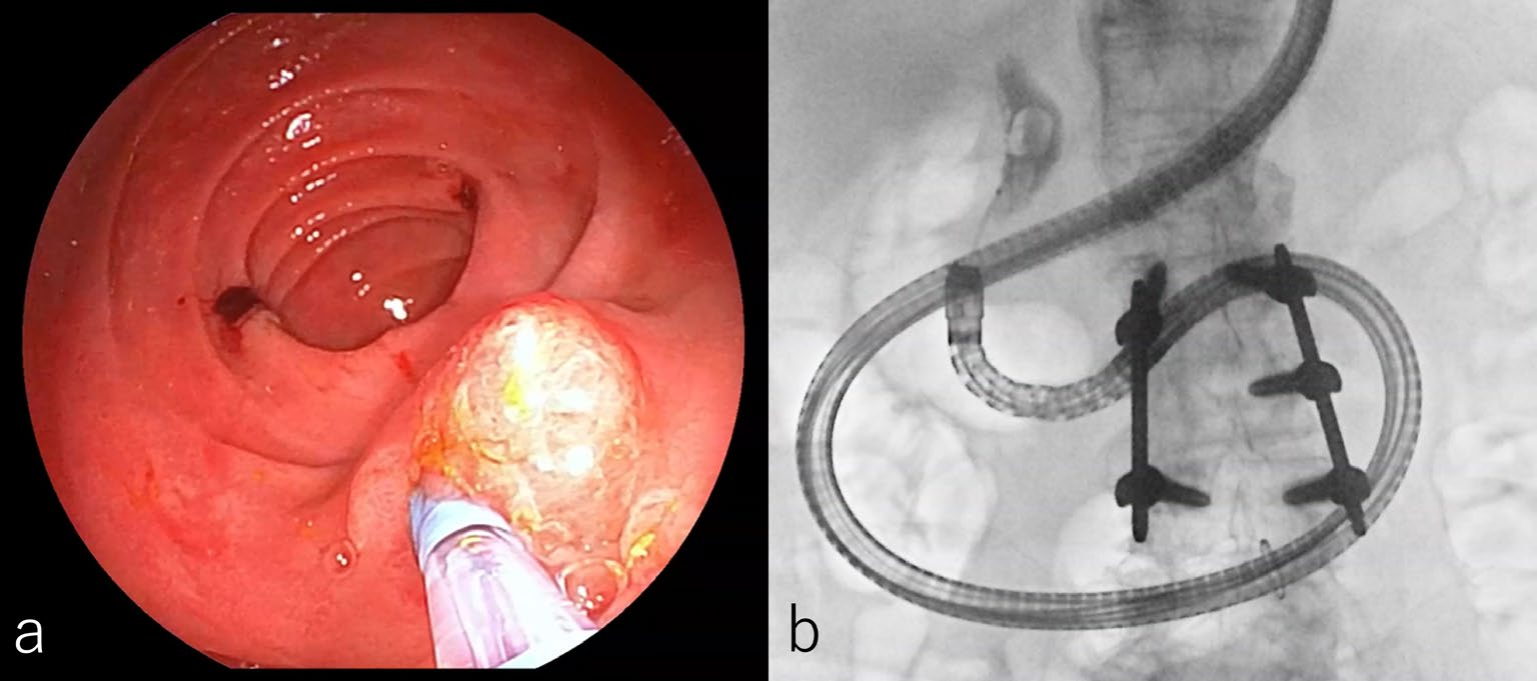
(a) X‐ray image and (b) endoscopic image of the T‐BAR method.

Keep the papilla in view, often at the 6 o'clock position [5]. Inflate the tip balloon for gentle fixation to the intestinal wall. Using the inflated balloon as an anchor/pivot, slowly advance the scope while applying down angulation to obtain a stable retroflex view without slipping into the blind end, while maintaining a working distance from the papilla (Figure 2). Standard biliary cannulation is then performed. The balloon is deflated, and treatment proceeds.

The T‐BAR method is a useful technique for optimizing biliary axis alignment during DBE‐ERCP in patients with an intact ligament of Treitz and native duodenal papilla. The T‐BAR technique can be applied regardless of overtube balloon position, scope loop formation, or the endoscopic orientation of the papilla. However, it is less suitable when the papilla is located close to the blind end, as this limits scope maneuverability.

## 3 Key Safety Points

Avoid excessive insufflation while the tip balloon is inflated to prevent blind‐limb overdistension, and use gentle, controlled scope manipulation to reduce the risk of perforation.

## Funding

The authors have nothing to report.

## Ethics Statement

The authors have nothing to report.

## Conflicts of Interest

The authors declare no conflicts of interest.

## Supporting information


**Video S1:** jhbp70094‐sup‐0001‐Supinfo.zip.

## Data Availability

The data that support the findings of this study are available on request from the corresponding author. The data are not publicly available due to privacy or ethical restrictions.
