# Supplementary material for: Tip‐Balloon Anchored Retroflex (T‐BAR) Method for Double Balloon Endoscopy‐Assisted ERCP in Roux‐en‐Y Anatomy
Source: J Hepatobiliary Pancreat Sci. 2026 Feb 23;33(6):e42–3. doi: 10.1002/jhbp.70094 (PMC13284809; doi:10.1002/jhbp.70094)
Supplement: Supplementary file 1 — Video S1: jhbp70094‐sup‐0001‐Supinfo.zip. [file JHBP-33-e42-s001.zip › jhbp70094-sup-0002-Supinfo2@Video text.docx]

Tip-Balloon Anchored Retroflex (T-BAR) method for Double balloon endoscopy-assisted ERCP in Roux-en-Y anatomy is demonstrated in this video.

First, we briefly explain the concept of the T-BAR method.

A typical retroflex approach involves gently pushing the scope with up angulation, but the tip may slip toward the blind end.

The T-BAR method inflates the tip balloon as an anchoring fulcrum, then gently pushes with up angulation to achieve stable retroflexion position.

Next, we present a representative case.

The patient is a man in his 70s with Roux-en-Y reconstruction after distal gastrectomy. Emergency DBE-ERCP was performed for common bile duct stones.

A double-balloon endoscope reached the papilla.

But the bile duct axis was nearly perpendicular to the catheter axis and selective biliary cannulation failed.

We attempted retroflex formation by slowly advancing the scope while applying down angulation under visualization;

however, the scope tip repeatedly slid toward the blind end, preventing stable positioning.

At this point, we perform the T-BAR method.

While keeping the papilla in view, Inflate the tip balloon for gentle fixation to the intestinal wall.

After confirming balloon inflation under fluoroscopy, Using the inflated balloon as an anchor, slowly advance the scope.

Applying down angulation to obtain a stable retroflex view without slipping into the blind end, while maintaining a working distance from the papilla.

Thereafter, biliary cannulation is performed.

After successful deep biliary access is confirmed, the tip balloon is deflated and subsequent therapeutic procedures are continued as usual.
